# Management of Ineffective Esophageal Hypomotility

**DOI:** 10.3389/fphar.2021.638915

**Published:** 2021-05-26

**Authors:** Sawangpong Jandee, Annelies Geeraerts, Hannelore Geysen, Nathalie Rommel, Jan Tack, Tim Vanuytsel

**Affiliations:** ^1^ Department of Chronic Diseases, Translational Research Center for Gastrointestinal Disorders (TARGID), Metabolism and Ageing (CHROMETA), KU Leuven, Leuven, Belgium; ^2^ Gastroenterology and Hepatology Unit, Department of Internal Medicine, Faculty of Medicine, Prince of Songkla University, Songkhla, Thailand; ^3^ Department of Neurosciences, Experimental Otorhinolaryngology, KU Leuven, Leuven, Belgium; ^4^ Department of Gastroenterology and Hepatology, Leuven University Hospitals, Leuven, Belgium

**Keywords:** esophageal hypomotility, ineffective esophageal motility, high resolution manometry, prokinetic, gastroesophageal reflux, dysphagia

## Abstract

Esophageal hypomotility in general and especially ineffective esophageal motility according to the Chicago criteria of primary motility disorders of the esophagus, is one of the most frequently diagnosed motility disorders on high resolution manometry and results in a large number of patients visiting gastroenterologists. Most patients with esophageal hypomotility present with gastroesophageal reflux symptoms or dysphagia. The clinical relevance of the motility pattern, however, is not well established but seems to be correlated with disease severity in reflux patients. The correlation with dysphagia is less clear. Prokinetic agents are commonly prescribed as first line pharmacologic intervention to target esophageal smooth muscle contractility and improve esophageal motor functions. However, the beneficial effects of these medications are limited and only confined to some specific drugs. Serotonergic agents, including buspirone, mosapride and prucalopride have been shown to improve parameters of esophageal motility although the effect on symptoms is less clear. Understanding on the complex correlation between esophageal hypomotility and esophageal symptoms as well as the limited evidence of prokinetic agents is necessary for physicians to appropriately manage patients with Ineffective Esophageal Motility (IEM).

## Introduction

High resolution manometry (HRM) is widely applied to evaluate esophageal motor function, resulting in a better recognition of esophageal motility disorders (Sweis et al., 2018). The most recently updated classification for esophageal motility disorders, the Chicago Classification version 3.0, was proposed in 2015 after two previous versions in 2008 and 2012. This classification was developed based on the analysis of clinical studies in healthy volunteers and patients, and categorized esophageal body motility disorders into major and minor disorders of peristalsis (Boland et al., 2016). Achalasia and major disorders of peristalsis, including distal esophageal spasm, jackhammer esophagus and absent contractility, reveal clinically relevant conditions for which evidence-based treatments are available—with the exception of absent contractility. This is in strong contrast with minor esophageal motility disorders, particularly ineffective esophageal motility (IEM), which still have unclear clinical implications and of which the management is not well established (Boland et al., 2016).

**FIGURE 1:**
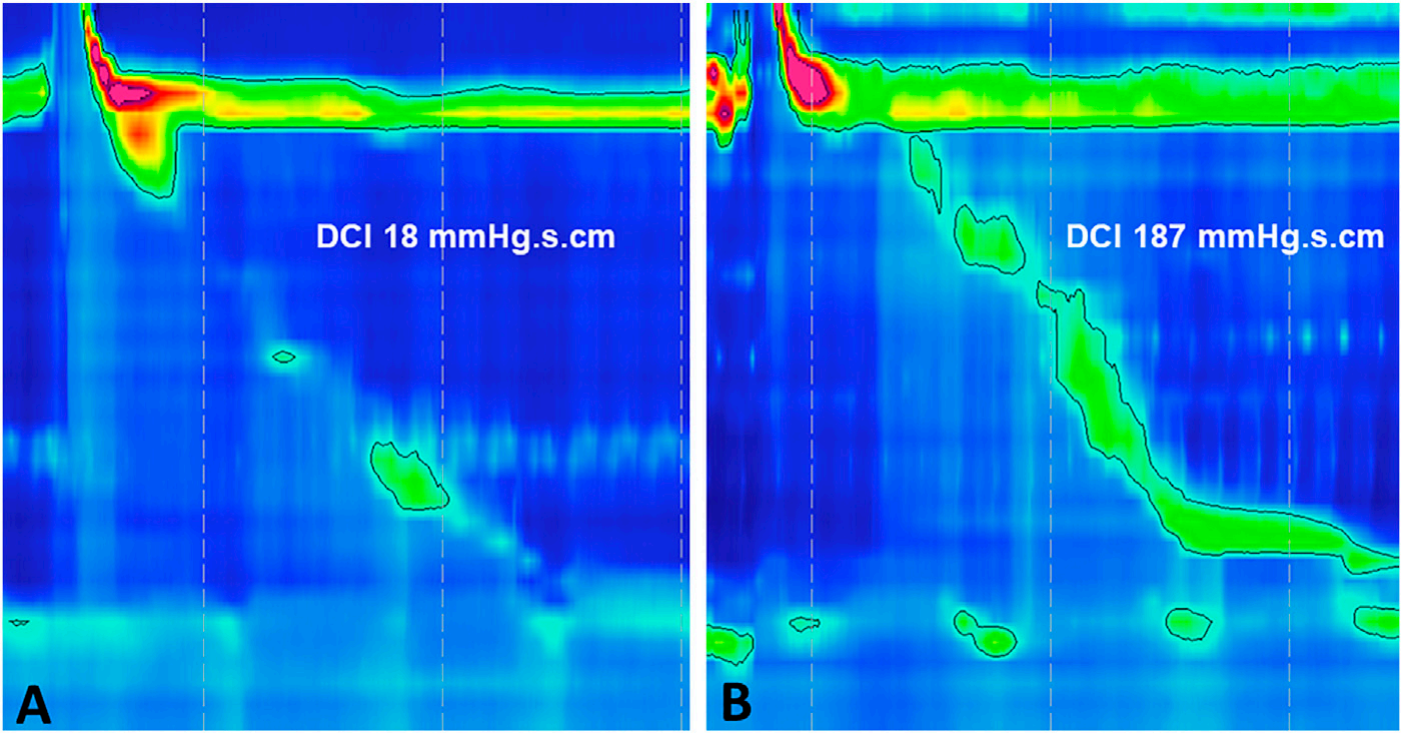
Esophageal high-resolution manometry demonstrating Ineffective Esophageal Motility (IEM) **(A)** failed peristalsis (DCI <100 mmHg s cm) and **(B)** weak peristalsis (DCI 100–450 mmHg s cm). IEM, ineffective esophageal motility; DCI, distal contractile integral.

IEM is reported in as many as 30% of patients undergoing HRM. It is defined by the Chicago classification as over 50% of swallows being either weak or failed [Distal Contractile Integral (DCI) ≤450 mmHg s cm], while normal lower esophageal sphincter relaxation is preserved [normal Integrated Relaxation Pressure (IRP)] (Figure 1) (Kahrilas et al., 2015).

In this narrative review, we summarize the available literature on the clinical associations of a manometric diagnosis of IEM and its treatment with prokinetics. A PubMed literature search was performed that included published articles in English through October 31, 2020 with combinations of the terms “ineffective esophageal motility,” “high resolution manometry,” “clinical relevance,” “pharmacological treatment,” and “prokinetic.” Reference lists of the retrieved articles were also searched for additional articles.

## Esophageal Hypomotility and Esophageal Symptoms

IEM is one of the most frequent findings on esophageal HRM. However, the association of esophageal hypomotility with symptoms is still controversial which makes this a confusing diagnostic entity. A prospective study of Hollenstein et al. in healthy volunteers revealed that as many as 17% of asymptomatic subjects demonstrated a pattern of IEM on routine esophageal manometry (Hollenstein et al., 2017). Moreover, IEM is detected in patients with a variety of esophageal symptoms, particularly gastroesophageal reflux symptoms and dysphagia, but these symptoms are not discriminative of IEM. A retrospective study from China evaluated 256 dysphagia patients who had unremarkable findings on esophagogastroscopy and underwent HRM. In this population IEM was the most common feature, in 38.6% of patients (Wang et al., 2019). However, several studies failed to demonstrate a correlation between IEM and esophageal symptoms (Xiao et al., 2014; Shetler et al., 2017). Indeed, proportions of heartburn, regurgitation, dysphagia, chest pain, and belching were similar in patients with and without IEM in observational studies (Min et al., 2015; Shetler et al., 2017). In addition, the correlation of the perception of dysphagia with abnormal bolus transit resulting from weak or absent peristalsis is also limited (Lazarescu et al., 2010; Roman et al., 2011).

IEM is more prevalent in smooth muscle disorders, such as scleroderma and other connective tissue disorders (Carlson et al., 2016). Moreover, phosphodiesterase inhibitors, calcium channel blockers and non-benzodiazepine antispasmodic agents can reduce esophageal contraction vigor and should be avoided in patients with esophageal hypomotility (Simren et al., 2003; Rangan et al., 2018).

## Esophageal Motor Dysfunction and Gastroesophageal Reflux Disease

Available data indicate that Gastroesophageal Reflux Disease (GERD) results from multiple predisposing factors in upper gastrointestinal motility, especially transient lower esophageal sphincter relaxations (TLESRs) (Schneider et al., 2010) which are more likely to be associated with reflux in GERD patients (Sifrim et al., 2001). In addition, esophageal body hypomotility is also more frequent in pH-monitoring proven GERD (Chan et al., 2011; Savarino et al., 2017).

Impaired esophageal clearance of the refluxate caused by ineffective primary and secondary peristalsis has also been illustrated in a higher proportion of patients with erosive esophagitis compared to a non-erosive reflux disease group (29 vs. 15%; *p* = 0.030) (Wu et al., 2007; Chen et al., 2014). Furthermore, the study of Wang et al. also demonstrated that erosive esophagitis and increasing GERD symptom severity are consistently associated with a greater likelihood of IEM, while the prevalence of IEM in non-erosive reflux disease and physiologic acid exposure is low (Wang et al., 2009). Additionally, severe IEM, defined as over 70% ineffective peristalsis, provides supportive evidence for a more severe GERD phenotype with an increased acid exposure in supine position (Shetler et al., 2017; Rengarajan et al., 2018). These data suggest a role for IEM in the pathophysiology of GERD. However, a case could also be made that esophageal hypomotility may result from increased reflux exposure. Longitudinal data, studying the sequence between GERD and esophageal hypomotility, are not available, but improvement of hypocontractility after anti-reflux surgery has been reported, suggesting that successful anti-reflux treatment may correct esophageal dysmotility (Munitiz et al., 2004). However, prolonged proton pump inhibitor (PPI) treatment in 23 patients with severe erosive esophagitis did not correct esophageal hypomotility (Xu et al., 2007). In a recent study on the reproducibility of esophageal manometric diagnoses, PPI treatment was not clearly associated with improved motility in esophageal hypomotility (Sandhu et al., 2020).

## Natural History and Prognosis

There is limited understanding of the natural history of IEM. However, IEM does not progress over time, and quality of life does not seem to be much impacted (Ravi et al., 2015). Patients with this minor esophageal motor abnormality reported minimal symptoms and needed few medical interventions during long-term follow-up over 6 years. Interestingly, the presence of peristaltic reserve by provocative maneuvers including multiple rapid swallowing (MRS) predicted a better prognosis and efficacy of prokinetics (Min et al., 2015; Gyawali et al., 2019).

## General Pharmacological Management

Pharmacologic interventions that are able to improve esophageal smooth muscle contractility or associated symptoms, are still limited and poorly effective (Smout and Fox, 2012). There is also no clear directive on when IEM needs management, as symptoms (e.g., dysphagia) and even GERD is not consistently identified with IEM. Therefore—unless GERD is identified—the decision if and how symptomatic patients with IEM should be treated, is challenging.

Diet, lifestyle modification and medical GERD management remain the cornerstone of therapy (Triadafilopoulos et al., 2016). Basically, acid-suppressive medication will treat reflux and reflux-related symptoms but it may not improve esophageal motor function (Xu et al., 2007; Sandhu et al., 2020). Besides PPI therapy, prokinetic agents are advised in GERD patients with esophageal motility disorders and PPI-refractory symptoms to enhance clearance of the refluxed contents (Scarpellini et al., 2016; Lin et al., 2019).

**FIGURE 2:**
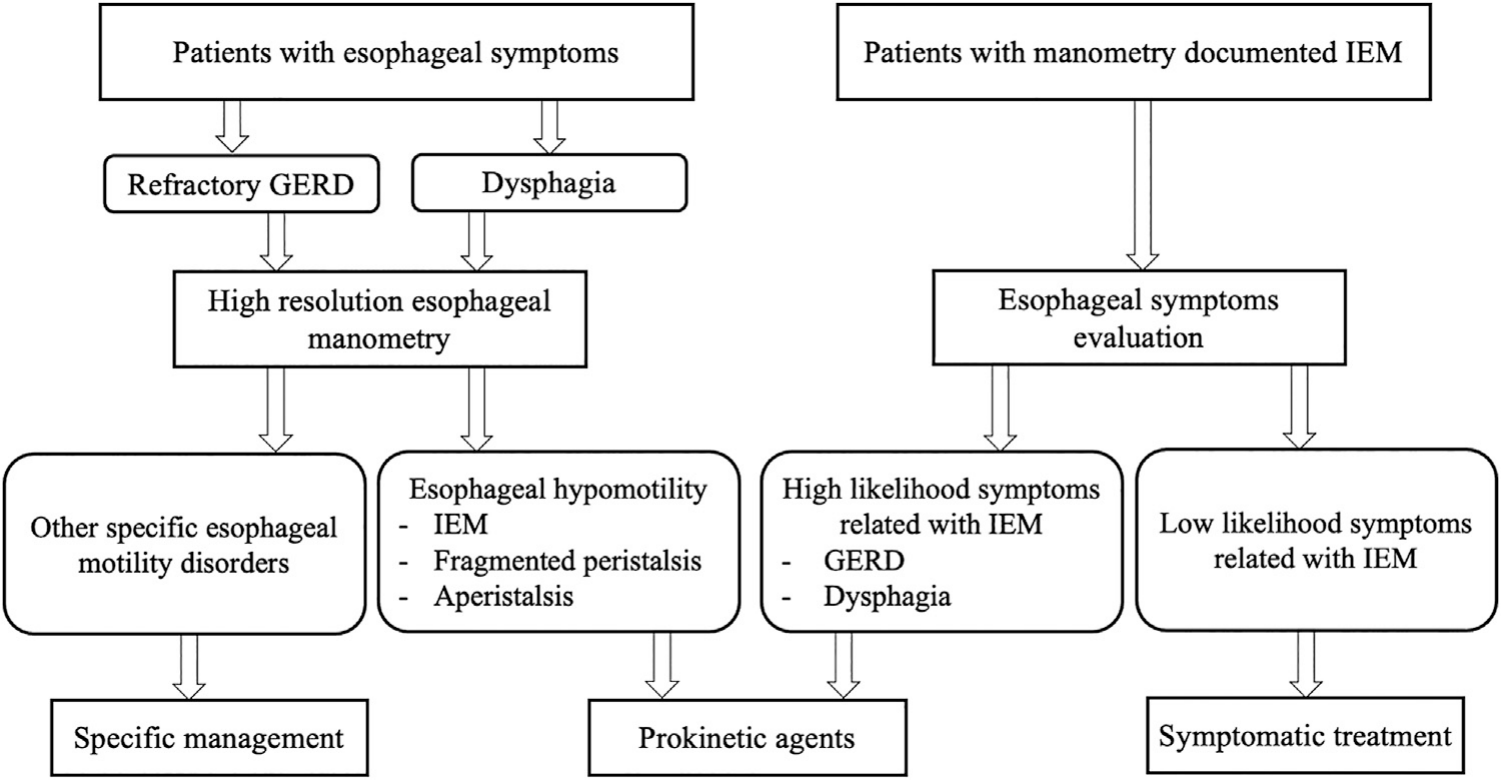
Algorithm for decision making on prokinetic prescription in patients with esophageal hypomotility. IEM, ineffective esophageal motility; GERD, gastroesophageal reflux disease.

Taking into account the limitations of IEM in terms of correlation to symptoms and GERD, we propose an algorithm to guide clinical decision making on prokinetic prescription in patients with esophageal hypomotility (Figure 2).

## Prokinetic Treatment

**FIGURE 3:**
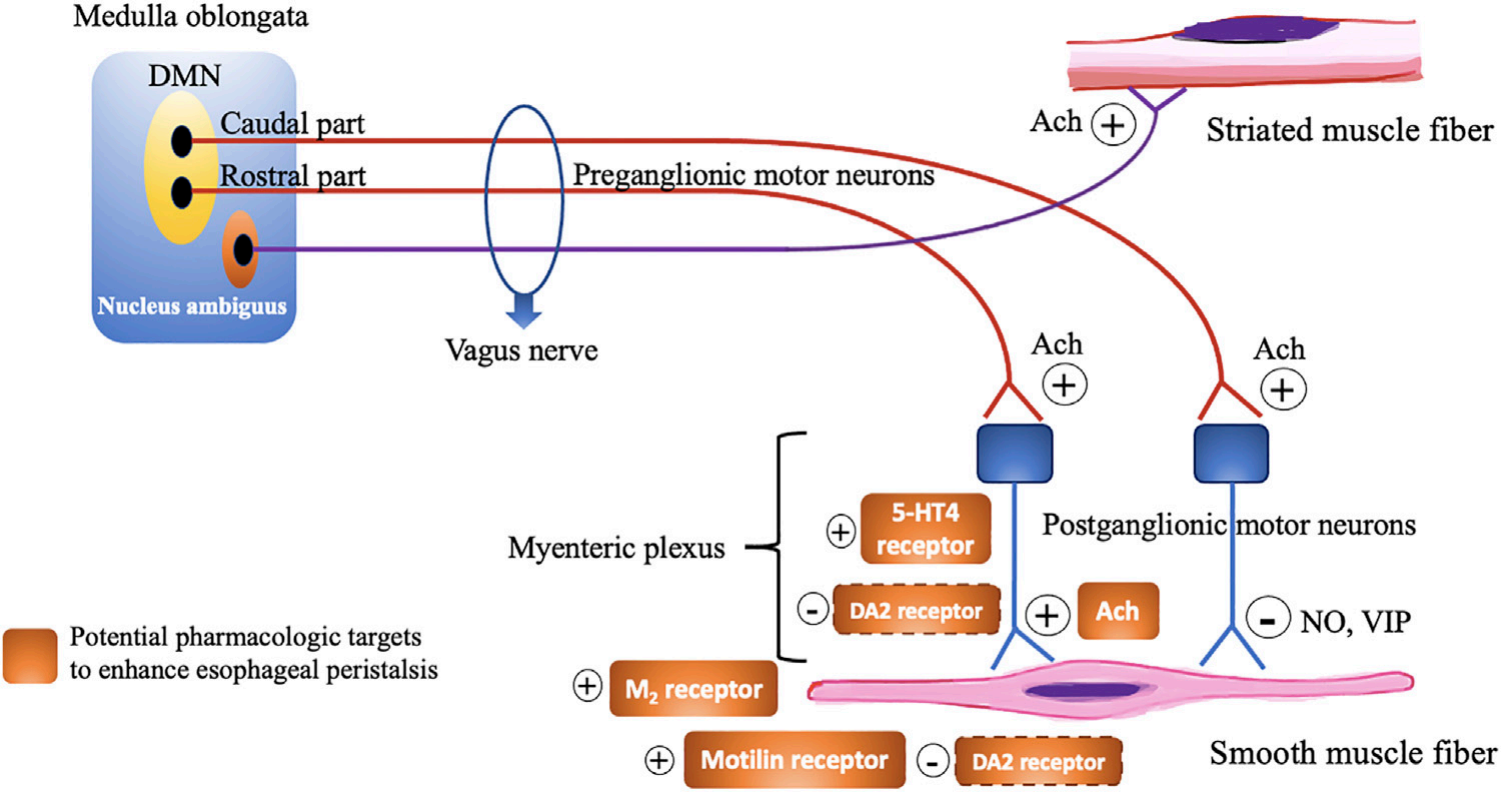
Schematic overview demonstrating the motor innervation of the esophagus and pharmacological targets to enhance esophageal peristalsis. Dashed line signify uncertainty of the exact receptor localization. DMN, Dorsal motor nucleus; Ach, Acetylcholine; NO, Nitric oxide; VIP, Vasoactive intestinal polypeptide; 5-HT, 5-Hydroxytryptamine; M, Muscarinic; DA, Dopamine.

**FIGURE 4:**
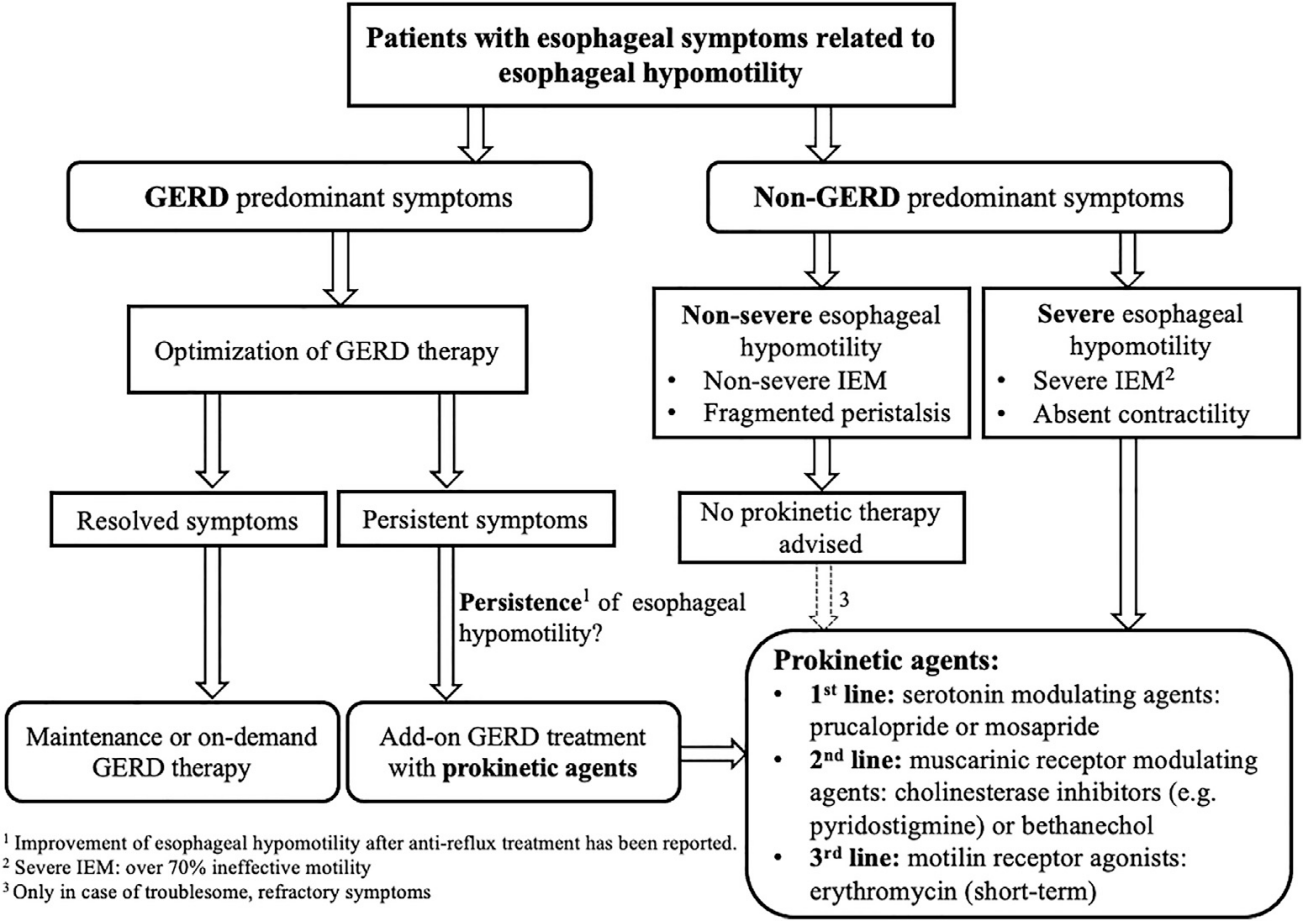
Guided algorithm illustrating prokinetic drugs application in setting of esophageal hypomotility GERD, Gastroesophageal reflux disease; IEM, Ineffective esophageal motility; 5-HT, 5-Hydroxytrptamine.

Esophageal peristalsis results from a concerted contraction and relaxation of circular and longitudinal musculature to propel the ingested food bolus toward the stomach. Peristalsis in the proximal esophagus, which is composed entirely of striated muscle, is dependent on central mechanisms, involving sequential activation of vagal lower motor neurons originating from the nucleus ambiguous (Kahrilas and Boeckxstaens, 2013). In contrast, distal esophageal peristalsis, which is mainly composed of smooth muscle fibers, is controlled by both central input, but mainly orchestrated by the ganglia of the enteric nervous system in the esophageal wall (Figure 3). There are two types of postganglionic myenteric motor neurons: excitatory neurons releasing the neurotransmitter acetylcholine (ACh), and inhibitory neurons that contain nitric oxide (NO) and vasoactive intestinal polypeptide (VIP). The balanced activation and interaction between these neurons and neurotransmitters are critical for the normal peristaltic function of the esophagus (Park and Conklin, 1999) and can be targeted by pharmacologic interventions, using prokinetics, in patients with severe esophageal hypomotility or absent contractility to restore esophageal motor function (Table 1; Figure 4). In patients with mild esophageal hypomotility we recommend a conservative approach taking into account the limited available clinical evidence and benign natural history. We also propose a hierarchy in the use of the available prokinetics based on the efficacy data and adverse events (Figure 4). In this overview we did not focus on cisapride and tegaserod because these drugs have been withdrawn from the market because of cardiovascular adverse events. Although tegaserod has been re-approved in 2019, the specific indication was limited to female patients with constipation predominant irritable bowel syndrome.

**TABLE 1:** Potentially beneficial prokinetic medications for esophageal hypomotility that have been studied in patients.

Prokinetic groups	Mechanism of action	Study design	Patients	Dose and duration	Outcome
Serotonergic agents					
- Buspirone Aggarwal et al. (2018)	5-HT1A agonist	Prospective, double-blind, placebo-controlled, crossover study	10 IEM/FD patients	10 mg before meals three times daily for 2 weeks	No difference in esophageal HRM parameters
- Mosapride Ruth et al. (1998)	5-HT4 agonist and weak 5-HT3 antagonist	Double-blind crossover trial	21 GERD patients	40 mg for 2 days	Decrease in total number of reflux episodes
Ruth et al. (2003)		Double-blind, randomized, double-dummy, three-way crossover study	41 GERD patients	30 mg three times daily for 7 days	- Small effects on peristaltic durations and amplitudes
					- No significant effect on the total number of esophageal contractions
Chen et al. (2013)		Prospective, double-blind, placebo-controlled, crossover study	18 IEM patients	40 mg single dose	Improved esophageal sensitivity of secondary peristalsis
- Prucalopride Lei et al. (2018)	High affinity and specificity for 5-HT4 agonist	Randomized placebo-controlled, crossover trial	15 GERD patients with IEM	4 mg single dose	- Increased peristaltic wave amplitude
					- Decreased threshold for triggering secondary peristalsis
- Sumatriptan Grossi et al. (2003)	5-HT1 agonist	Prospective, double-blind, placebo-controlled, crossover study^40^	10 IEM patients with chest pain and dysphagia	6 mg subcutaneous in the morning and afternoon (two doses)	- Increased number of swallows
					- Increased number of primary esophageal motor waves
Motilin receptor agonists					
- Erythromycin Chrysos et al. (2001)		Randomized single-blind study	15 GERD patients	200 mg IV single dose	Increased amplitude, duration, velocity and strength of esophageal peristalsis
Chang et al. (2003)		Single arm study	45 DM patients	Oral 250 mg three times daily for 2 weeks	Shorter esophageal transit time
Tsai et al. (1995)		Single arm study	15 DM patients	- Oral form for 2 weeks	- Shorter esophageal transit time
				- Dose not specified	- Less esophageal residue
Muscarinic receptor agonists					
- Bethanechol Agrawal et al. (2007)		Single arm, interpreter blinded study	Seven severe IEM patients	50 mg orally	- Improved contraction pressures
					- Improved distal esophageal amplitude
					- Enhanced complete bolus transit of the esophagus
Grevenitis et al. (2012)		Retrospective chart review	26 IEM patients	25 mg three times daily for an average of 7 months	50% improvement in dysphagia

5-HT, 5-hydroxytryptamine; IEM, ineffective esophageal motility; FD, functional dysphagia; HRM, high resolution manometry; GERD, gastroesophageal reflux disease; IV, intravenous; DM, diabetes mellitus.

### Dopamine 2 Receptor Blockers

Metoclopramide augmented esophageal contraction in healthy volunteers (Mikami et al., 2016) but in IEM, this conventional prokinetic agent is not beneficial (Gyawali et al., 2019). The acute effects of oral metoclopramide (40 mg/day) and domperidone (80 mg/day) on esophageal motor activity and acid reflux has been assessed in a randomized, double-blind, placebo-controlled study in 20 patients with erosive esophagitis. Both drugs caused a significant increase in lower esophageal sphincter (LES) pressure. However, neither esophageal body motility nor duration of esophageal acid exposure were affected by the prokinetics in comparison to placebo (Grande et al., 1992). Both domperidone (QT prolongation) and metoclopramide (extrapyramidal manifestations) have been associated with relevant adverse events, which are potentially serious (Leelakanok et al., 2016; Svendsen et al., 2018). In view of the lack of substantial efficacy they should probably not be used when attempting to treat esophageal hypomotility.

### Serotonin Modulating Agents

#### Buspirone

Buspirone, an anxiolytic drug, is a partial agonist for 5-HT (hydroxytryptamine) 1A receptors, as well as an antagonist for dopamine D2 autoreceptors, with some evidence of a weak agonistic effect on 5-HT2 receptors (Loane and Politis, 2012). In the enteric nervous system, 5-HT1A receptors activation can release ACh from the nerve terminals and then stimulate esophageal motor function by muscarinic receptors on smooth muscle cells. (Eduard et al., 2017). Buspirone has been shown to augment esophageal peristaltic amplitude in healthy volunteers. Blonski et al. and Di Stefano et al. administered 20 mg of buspirone to healthy adults and measured esophageal motility by conventional manometry within 60 min of administration in a blinded, placebo-controlled trial. The mean distal esophageal amplitude and duration were increased in both studies after a single dose of buspirone (Blonski et al., 2009; Di Stefano et al., 2012).

In systemic sclerosis (SSc), Karamanolis et al. evaluated 21 consecutive symptomatic patients with esophageal hypomotility, using a one-time dose of 10 mg buspirone compared to 10 mg domperidone. They found an increased resting lower esophageal sphincter (LES) pressure after buspirone, but no other significant change in esophageal peristalsis (Karamanolis et al., 2015). Another study of the same group, showed the same results in a non-randomized open-label trial of 4 weeks of 20 mg buspirone dosage in 22 SSc patients (Karamanolis et al., 2016). However, patients with SSc are a specific group within the spectrum of esophageal hypomotility and in many patients, esophageal manometry will show absent contractility rather than IEM. Even if the data are limited, the pathophysiological mechanisms underlying esophageal dysmotility in SSc are probably due to a complex interplay of vascular, immune, and neural abnormalities. Pharmacological therapy may provide some benefit in neuropathic and myopathic dysfunction, while it will most likely not be efficient in later stages of fibrosis (Suto and Czirjak, 2009; Scheerens et al., 2015).

Recently, buspirone was studied in IEM and functional dysphagia patients, but there was no statistically significant difference in the high resolution esophageal parameters measured, as well as symptom outcomes compared to placebo (Table 1) (Aggarwal et al., 2018).

#### Mosapride

Mosapride is a 5-HT4 receptor agonist, and its metabolites also have a weak 5-HT3 antagonistic effect. Mosapride has no affinity for 5-HT1, 5-HT2 or dopamine D2 receptors. In 20 asymptomatic volunteers, mosapride 3 mg t.i.d. for 3 days increased the rate of complete bolus transit and accelerated esophageal bolus transit in a randomized double-blind crossover design (Cho et al., 2006). It has also been demonstrated that a single 40 mg dose of mosapride increased the likelihood of secondary peristaltic responses to abrupt intra-esophageal air distension (Chen et al., 2011). Another study in nine healthy volunteers by Fukazawa et al. (2014) revealed that mosapride 40 mg augmented peristaltic contractions, especially in the distal esophageal segments (Fukazawa et al., 2014).

In GERD patients, mosapride was significantly more effective than placebo in decreasing the total number of reflux episodes (Ruth et al., 1998). Moreover, a high dose of mosapride (90 mg/day) has been reported to improve esophageal motor function and acid reflux parameters (Ruth et al., 2003).

In IEM patients, a single high-dose of mosapride (40 mg) decreased the threshold volume of secondary peristalsis during rapid air distension compared with placebo, but had limited effect on the motor properties (Chen et al., 2013). A partial effect of mosapride on esophageal motility has only been shown with high dose, in contrast to low or standard dose (15 mg/day) which did not change the esophageal motility parameters (Koshino et al., 2010).

#### Prucalopride

Prucalopride is an enterokinetic agent which acts by facilitating the release of ACh from neurons of the myenteric plexus *via* a high affinity and specificity for 5-HT4 receptors (Briejer et al., 2001). Because of its highly specific effect on the 5-HT4 receptor in the absence of affinity for the hERG cardiac potassium channel, no cardiac toxicity has been reported in contrast to older 5-HT4 agonists, including cisapride (De Maeyer et at., 2008; Tack et al., 2012). Acute administration of 4 mg prucalopride enhanced mechanosensitivity of distension-induced secondary peristalsis and promoted esophageal contractility in 11 healthy adults (Yi et al., 2016). Kessing et al. demonstrated that 4 mg-prucalopride for 6 days in 21 healthy subjects reduced esophageal acid exposure and accelerated gastric emptying without significant effects on esophageal motility (Kessing et al., 2014).

In GERD patients with IEM, a single-dose of prucalopride enhanced primary and secondary peristalsis. The threshold volume for triggering secondary peristalsis during slow and rapid injection of air into esophagus was decreased, with limited impact on secondary peristaltic amplitude (Lei et al., 2018).

#### Sumatriptan

Sumatriptan is a 5-HT1D receptor agonist used in the treatment of migraine. In 3–5% of the patients this medication triggered chest symptoms which were hypothesized to originate from esophageal hypercontractility (Brown et al., 1991). In 16 healthy volunteers, one subcutaneous injection of 6 mg of sumatriptan significantly altered esophageal motor function with higher amplitude of esophageal contractions (Foster et al., 1999).

In patients with dysphagia and chest pain with IEM on manometry, a subcutaneous injection of sumatriptan increased the number of swallows and primary peristaltic waves, but not the amplitude or propagation velocity of esophageal motility (Grossi et al., 2003).

### Motilin Receptor Agonists

#### Erythromycin

Erythromycin is an old macrolide antibiotic with prokinetic properties. The prokinetic action of erythromycin has been mainly attributed to its property of activating motilin receptors on smooth muscle fibers (Sanger et al., 2013). Its prokinetic efficacy was studied in GERD patients with a significant increase of the amplitude, duration, velocity and strength of esophageal peristalsis after a single intravenously administered dose (Chrysos et al., 2001).

Furthermore, oral erythromycin improved esophageal and gastric motility in diabetic patients and also resulted in a better control of blood sugar. The esophageal transit time, evaluated by radionuclide labeled liquid and solid meals, was significantly shorter (Chang et al., 2003). Another study from Taiwan reported an improvement of esophageal hypomotility in 15 diabetic patients, as evaluated by a non-invasive radionuclide esophageal transit test (Tsai et al., 1995). Despite a possible benefit on esophageal motility, disadvantages, including the risk of inducing microbial resistance, tachyphylaxis and cardiac dysrhythmia (QTc prolongation), should be taken into consideration (Goossens et al., 2005; Ray, et al., 2004).

### Muscarinic Receptor Modulating Agents

#### Bethanechol

Bethanechol, a direct-acting muscarinic receptor agonist, has been used in the past as a promotility agent for treating GERD. This drug acts by mimicking the effect of ACh directly at the postganglionic cholinergic receptors, and has been shown to increase the LES pressure and improve esophageal peristaltic pressures in healthy volunteers (Ramirez and Richter, 1993).

In patients with severe IEM, oral bethanechol has been shown to significantly improve contraction pressures, distal esophageal amplitude and complete bolus transit of the esophagus (Agrawal et al., 2007). A retrospective chart review of 26 patients with a known diagnosis of IEM who were treated with bethanechol at the esophageal disorders clinic, also reported a positive response, defined as improvement of dysphagia, in 50% of patients (Grevenitis et al., 2012). However, more than a quarter of patients discontinued the treatment due to intolerable cholinergic side effects, including nausea, somnolence and increased urinary frequency.

## Conclusion and Future Direction

It is important to emphasize that esophageal hypomotility is a manometric diagnosis that can be seen in healthy asymptomatic individuals and does not necessarily have a clear relevance in esophageal symptoms. Prokinetic agents can be considered in patients with esophageal symptoms thought to originate from IEM. However, currently available conventional prokinetic agents (mainly dopamine-2 antagonists) have not shown ability to restore the esophageal motor function in IEM. The potentially beneficial pharmacological agents are confined to specific serotonergic agents and motilin receptor agonists, but the scientific evidence is limited and larger future studies with a double-blind, randomized controlled design potentially including simultaneously impedance monitoring for bolus flow are needed to clearly identify its efficacy and clinical implication in patients with esophageal hypomotility.

## References

[B1] Aggarwal N. Thota P. N. Lopez R. Gabbard S. (2018). A Randomized Double-Blind Placebo-Controlled Crossover-Style Trial of Buspirone in Functional Dysphagia and Ineffective Esophageal Motility. Neurogastroenterol. Motil. 30, e13213. 10.1111/nmo.13213 28884884

[B2] Agrawal A. Hila A. Tutuian R. Mainie I. Castell D. O. (2007). Bethanechol Improves Smooth Muscle Function in Patients with Severe Ineffective Esophageal Motility. J. Clin. Gastroenterol. 41, 366–370. 10.1097/01.mcg.0000225542.03880.68 17413603

[B3] Blonski W. Vela M. F. Freeman J. Sharma N. Castell D. O. (2009). The Effect of Oral Buspirone, Pyridostigmine, and Bethanechol on Esophageal Function Evaluated with Combined Multichannel Esophageal Impedance-Manometry in Healthy Volunteers. J. Clin. Gastroenterol. 43, 253–260. 10.1097/mcg.0b013e318167b89d 18987553

[B4] Boland K. Abdul-Hussein M. Tutuian R. Castell D. O. (2016). Characteristics of Consecutive Esophageal Motility Diagnoses after a Decade of Change. J. Clin. Gastroenterol. 50, 301–306. 10.1097/mcg.0000000000000402 26422715

[B5] Briejer M. R. Bosmans J.-P. Van Daele P. Jurzak M. Heylen L. Leysen J. E. (2001). The *In Vitro* Pharmacological Profile of Prucalopride, a Novel Enterokinetic Compound. Eur. J. Pharmacol. 423, 71–83. 10.1016/s0014-2999(01)01087-1 11438309

[B6] Brown E. G. Endersby C. A. Smith R. N. Talbot J. C. C. (1991). The Safety and Tolerability of Sumatriptan: an Overview. Eur. Neurol. 31, 339–344. 10.1159/000116762 1653142

[B7] Carlson D. A. Crowell M. D. Kimmel J. N. Patel A. Gyawali C. P. Hinchcliff M. (2016). Loss of Peristaltic reserve, Determined by Multiple Rapid Swallows, Is the Most Frequent Esophageal Motility Abnormality in Patients with Systemic Sclerosis. Clin. Gastroenterol. Hepatol. 14, 1502–1506. 10.1016/j.cgh.2016.03.039 27062902 PMC5028229

[B8] Chan W. W. Haroian L. R. Gyawali C. P. (2011). Value of Preoperative Esophageal Function Studies before Laparoscopic Antireflux Surgery. Surg. Endosc. 25, 2943–2949. 10.1007/s00464-011-1646-9 21424193

[B9] Chang C.-T. Shiau Y.-C. Lin C.-C. Li T.-C. Lee C.-C. Kao C.-H. (2003). Improvement of Esophageal and Gastric Motility after 2-week Treatment of Oral Erythromycin in Patients with Non-insulin-dependent Diabetes Mellitus. J. Diabetes its Complications. 17, 141–144. 10.1016/s1056-8727(02)00168-x 12738398

[B10] Chen C. L. Liu T. T. Yi C. H. Orr W. C. (2011). Effects of Mosapride on Esophageal Secondary Peristalsis in Humans. Neurogastroenterol. Motil. 23, 606–e249. 10.1111/j.1365-2982.2011.01714.x 21501334

[B11] Chen C.-L. Yi C.-H. Liu T.-T. Orr W. C. (2013). Effects of Mosapride on Secondary Peristalsis in Patients with Ineffective Esophageal Motility. Scand. J. Gastroenterol. 48, 1363–1370. 10.3109/00365521.2013.840856 24099237

[B12] Chen C.-L. Yi C.-H. Liu T.-T. (2014). Relevance of Ineffective Esophageal Motility to Secondary Peristalsis in Patients with Gastroesophageal Reflux Disease. J. Gastroenterol. Hepatol. 29, 296–300. 10.1111/jgh.12367 23981079

[B13] Cho Y. K. Choi M.-G. Han H. W. Park J. M. Oh J. H. Jeong J. J. (2006). The Effect of Mosapride on Esophageal Motility and Bolus Transit in Asymptomatic Volunteers. J. Clin. Gastroenterol. 40, 286–292. 10.1097/01.mcg.0000210103.82241.97 16633098

[B14] Chrysos E. Tzovaras G. Epanomeritakis E. Tsiaoussis J. Vrachasotakis N. Vassilakis J. S. (2001). Erythromycin Enhances Oesophageal Motility in Patients with Gastro-Oesophageal Reflux. ANZ J. Surg. 71, 98–102. 10.1046/j.1440-1622.2001.02005.x 11413601

[B15] De Maeyer J. H. Lefebvre R. A. Schuurkes J. A. J. (2008). 5-HT4 Receptor Agonists: Similar but Not the Same. Neurogastroenterol. Motil. 20, 99–112. 10.1111/j.1365-2982.2007.01059.x 18199093

[B17] Di Stefano M. Papathanasopoulos A. Blondeau K. Vos R. Boecxstaens V. Farré R. (2012). Effect of Buspirone, a 5-HT1A Receptor Agonist, on Esophageal Motility in Healthy Volunteers. Dis. Esophagus. 25, 470–476. 10.1111/j.1442-2050.2011.01275.x 22050410

[B18] Eduard L. Golubev Y. Puzikov A. (2017). Serotonin Receptors Mediate Contractile Activity of Rat's Esophagus *In-Vivo* . Arch. Organ. Transpl. 2, 019–022. 10.17352/2640-7973.000007

[B19] Foster J. M. Houghton L. A. Whorwell P. J. Morris J. (1999). Altered Oesophageal Motility Following the Administration of the 5-HT1 Agonist, Sumatriptan. Aliment. Pharmacol. Ther. 13, 927–936. 10.1046/j.1365-2036.1999.00518.x 10383528

[B20] Fukazawa K. Furuta K. Adachi K. Moritou Y. Saito T. Kusunoki R. (2014). Effects of Mosapride on Esophageal Motor Activity and Esophagogastric junction Compliance in Healthy Volunteers. J. Gastroenterol. 49, 1307–1313. 10.1007/s00535-013-0876-0 24013654

[B21] Goossens H. Ferech M. Vander Stichele R. Elseviers M. (2005). Outpatient Antibiotic Use in Europe and Association with Resistance: a Cross-National Database Study. The Lancet. 365, 579–587. 10.1016/s0140-6736(05)17907-0 15708101

[B22] Grande L. Lacima G. Ros E. Garcia-Valdecasas J. C. Fuster J. Visa J. (1992). Lack of Effect of Metoclopramide and Domperidone on Esophageal Peristalsis and Esophageal Acid Clearance in Reflux Esophagitis. Dig. Dis Sci. 37, 583–588. 10.1007/bf01307583 1551349

[B23] Grevenitis P. Rife C. Castell D. (2012). Evidence that Bethanechol May Improve Dysphagia in Patients with Ineffective Esophageal Motility. Am. J. Gastroenterol. 107, S11. 10.14309/00000434-201210001-00024

[B24] Grossi L. Ciccaglione A. F. Marzio L. (2003). Effect of the 5-HT1 Agonist Sumatriptan on Oesophageal Motor Pattern in Patients with Ineffective Oesophageal Motility. Neurogastroenterol Motil. 15, 9–14. 10.1046/j.1365-2982.2003.00380.x 12588464

[B25] Gyawali C. P. Sifrim D. Carlson D. A. Hawn M. Katzka D. A. Pandolfino J. E. (2019). Ineffective Esophageal Motility: Concepts, Future Directions, and Conclusions from the Stanford 2018 Symposium. Neurogastroenterology Motil. 31, e13584. 10.1111/nmo.13584 PMC9380027 30974032

[B26] Hollenstein M. Thwaites P. Bütikofer S. Heinrich H. Sauter M. Ulmer I. (2017). Pharyngeal Swallowing and Oesophageal Motility during a Solid Meal Test: a Prospective Study in Healthy Volunteers and Patients with Major Motility Disorders. Lancet Gastroenterol. Hepatol. 2, 644–653. 10.1016/s2468-1253(17)30151-6 28684261

[B27] Kahrilas P. J. Boeckxstaens G. (2013). The Spectrum of Achalasia: Lessons from Studies of Pathophysiology and High-Resolution Manometry. Gastroenterology. 145, 954–965. 10.1053/j.gastro.2013.08.038 23973923 PMC3835179

[B28] Kahrilas P. J. Bredenoord A. J. Fox M. Gyawali C. P. Roman S. Smout A. J. P. M. (2015). The Chicago Classification of Esophageal Motility Disorders, v3.0. Neurogastroenterol. Motil. 27, 160–174. 10.1111/nmo.12477 25469569 PMC4308501

[B29] Karamanolis G. P. Panopoulos S. Denaxas K. Karlaftis A. Zorbala A. Kamberoglou D. (2016). The 5-HT1A Receptor Agonist Buspirone Improves Esophageal Motor Function and Symptoms in Systemic Sclerosis: a 4-week, Open-Label Trial. Arthritis Res. Ther. 18, 195. 10.1186/s13075-016-1094-y 27586891 PMC5009650

[B30] Karamanolis G. P. Panopoulos S. Karlaftis A. Denaxas K. Kamberoglou D. Sfikakis P. P. (2015). Beneficial Effect of the 5‐HT 1A Receptor Agonist Buspirone on Esophageal Dysfunction Associated with Systemic Sclerosis: A Pilot Study. United Eur. Gastroenterol. j. 3, 266–271. 10.1177/2050640614560453 PMC4480533 26137301

[B31] Kessing B. F. Smout A. J. P. M. Bennink R. J. Kraaijpoel N. Oors J. M. Bredenoord A. J. (2014). Prucalopride Decreases Esophageal Acid Exposure and Accelerates Gastric Emptying in Healthy Subjects. Neurogastroenterol. Motil. 26, 1079–1086. 10.1111/nmo.12359 24891067

[B32] Koshino K. Adachi K. Furuta K. Ohara S. Morita T. Nakata S. (2010). Effects of Mosapride on Esophageal Functions and Gastroesophageal Reflux. J. Gastroenterol. Hepatol. 25, 1066–1071. 10.1111/j.1440-1746.2010.06280.x 20594220

[B33] Lazarescu A. Karamanolis G. Aprile L. De Oliveira R. B. Dantas R. Sifrim D. (2010). Perception of Dysphagia: Lack of Correlation with Objective Measurements of Esophageal Function. Neurogastroenterol. Motil. 22, 1292–e337. 10.1111/j.1365-2982.2010.01578.x 20718946

[B34] Leelakanok N. Holcombe A. Schweizer M. L. (2016). Domperidone and Risk of Ventricular Arrhythmia and Cardiac Death: A Systematic Review and Meta-Analysis. Clin. Drug Investig. 36, 97–107. 10.1007/s40261-015-0360-0 26649742

[B35] Lei W.-Y. Hung J.-S. Liu T.-T. Yi C.-H. Chen C.-L. (2018). Influence of Prucalopride on Esophageal Secondary Peristalsis in Reflux Patients with Ineffective Motility. J. Gastroenterol. Hepatol. 33, 650–655. 10.1111/jgh.13986 28898473

[B36] Lin S. Li H. Fang X. (2019). Esophageal Motor Dysfunctions in Gastroesophageal Reflux Disease and Therapeutic Perspectives. J. Neurogastroenterol. Motil. 25, 499–507. 10.5056/jnm19081 31587540 PMC6786454

[B37] Loane C. Politis M. (2012). Buspirone: what Is it All about?. Brain Res. 1461, 111–118. 10.1016/j.brainres.2012.04.032 22608068

[B38] Mikami H. Ishimura N. Fukazawa K. Okada M. Izumi D. Shimura S. (2016). Effects of Metoclopramide on Esophageal Motor Activity and Esophagogastric Junction Compliance in Healthy Volunteers. J. Neurogastroenterol. Motil. 22, 112–117. 10.5056/jnm15130 26507875 PMC4699728

[B39] Min Y. W. Shin I. Son H. J. Rhee P.-L. (2015). Multiple Rapid Swallow Maneuver Enhances the Clinical Utility of High-Resolution Manometry in Patients Showing Ineffective Esophageal Motility. Medicine. 94, e1669. 10.1097/MD.0000000000001669 26448010 PMC4616734

[B40] Munitiz V. Ortiz A. Martinez de Haro L. F. Molina J. Parrilla P. (2004). Ineffective Oesophageal Motility Does Not Affect the Clinical Outcome of Open Nissen Fundoplication. Br. J. Surg. 91, 1010–1014. 10.1002/bjs.4597 15286963

[B41] Park H. Conklin J. L. (1999). Neuromuscular Control of Esophageal Peristalsis. Curr. Gastroenterol. Rep. 1, 186–197. 10.1007/s11894-999-0033-3 10980948

[B42] Ramirez B. Richter J. E. (1993). Review Article: Promotility Drugs in the Treatment of Gastro-Oesophageal Reflux Disease. Aliment. Pharmacol. Ther. 7, 5–20. 10.1111/j.1365-2036.1993.tb00064.x 8094981

[B43] Rangan V. George N. S. Khan F. Geng Z. Gabbard S. Kichler A. (2018). Severity of Ineffective Esophageal Motility Is Associated with Utilization of Skeletal Muscle Relaxant Medications. Neurogastroenterol. Motil. 30, e13235. 10.1111/nmo.13235 29027725

[B44] Ravi K. Friesen L. Issaka R. Kahrilas P. J. Pandolfino J. E. (2015). Long-term Outcomes of Patients with Normal or Minor Motor Function Abnormalities Detected by High-Resolution Esophageal Manometry. Clin. Gastroenterol. Hepatol. 13, 1416–1423. 10.1016/j.cgh.2015.02.046 25771245 PMC4510014

[B45] Ray W. A. Murray K. T. Meredith S. Narasimhulu S. S. Hall K. Stein C. M. (2004). Oral Erythromycin and the Risk of Sudden Death from Cardiac Causes. N. Engl. J. Med. 351, 1089–1096. 10.1056/nejmoa040582 15356306

[B46] Rengarajan A. Bolkhir A. Gor P. Wang D. Munigala S. Gyawali C. P. (2018). Esophagogastric junction and Esophageal Body Contraction Metrics on High-Resolution Manometry Predict Esophageal Acid burden. Neurogastroenterol. Motil. 30, e13267. 10.1111/nmo.13267 29266647

[B47] Roman S. Lin Z. Kwiatek M. A. Pandolfino J. E. Kahrilas P. J. (2011). Weak Peristalsis in Esophageal Pressure Topography: Classification and Association with Dysphagia. Am. J. Gastroenterol. 106, 349–356. 10.1038/ajg.2010.384 20924368 PMC3035759

[B48] Ruth M. Finizia C. Cange L. Lundell L. (2003). The Effect of Mosapride on Oesophageal Motor Function and Acid Reflux in Patients with Gastro-Oesophageal Reflux Disease. Eur. J. Gastroenterol. Hepatol. 15, 1115–1121. 10.1097/00042737-200310000-00009 14501621

[B49] Ruth M. Hamelin B. Röhss K. Lundell L. (1998). The Effect of Mosapride, a Novel Prokinetic, on Acid Reflux Variables in Patients with Gastro-Oesophageal Reflux Disease. Aliment. Pharmacol. Ther. 12, 35–40. 10.1046/j.1365-2036.1998.00268.x 9692698

[B50] Sandhu A. Eisa M. Yamasaki T. Shibli F. Fass R. (2020). Durability of Esophageal Motor Disorders Identified on High-Resolution Esophageal Manometry: A Case Series. Adv. Ther. 37, 2560–2571. 10.1007/s12325-020-01326-w 32285339 PMC7467470

[B51] Sanger G. J. Wang Y. Hobson A. Broad J. (2013). Motilin: towards a New Understanding of the Gastrointestinal Neuropharmacology and Therapeutic Use of Motilin Receptor Agonists. Br. J. Pharmacol. 170, 1323–1332. 10.1111/bph.12075 23189978 PMC3838679

[B52] Savarino E. Bredenoord A. J. Bredenoord A. J. Fox M. Pandolfino J. E. Roman S. (2017). Advances in the Physiological Assessment and Diagnosis of GERD. Nat. Rev. Gastroenterol. Hepatol. 14, 665–676. 10.1038/nrgastro.2017.130 28951582

[B53] Scarpellini E. Ang D. Pauwels A. De Santis A. Vanuytsel T. Tack J. (2016). Management of Refractory Typical GERD Symptoms. Nat. Rev. Gastroenterol. Hepatol. 13, 281–294. 10.1038/nrgastro.2016.50 27075264

[B54] Scheerens C. Tack J. Rommel N. (2015). Buspirone, a New Drug for the Management of Patients with Ineffective Esophageal Motility?. United Eur. Gastroenterol. j. 3, 261–265. 10.1177/2050640615585688 PMC4480541 26137300

[B55] Schneider J. H. Küper M. A. Königsrainer A. Brücher B. L. D. M. (2010). Transient Lower Esophageal Sphincter Relaxation and Esophageal Motor Response. J. Surg. Res. 159, 714–719. 10.1016/j.jss.2009.02.021 19577763

[B56] Shetler K. P. Bikhtii S. Triadafilopoulos G. (2017). Ineffective Esophageal Motility: Clinical, Manometric, and Outcome Characteristics in Patients with and without Abnormal Esophageal Acid Exposure. Dis. Esophagus. 30, 1–8. 10.1093/dote/dox012 28475749

[B57] Sifrim D. Holloway R. Silny J. Tack J. Lerut A. Janssens J. (2001). Composition of the Postprandial Refluxate in Patients with Gastroesophageal Reflux Disease. Am. J. Gastroenterol. 96, 647–655. 10.1111/j.1572-0241.2001.03598.x 11280529

[B58] Simren M. Silny J. Holloway R. Tack J. Janssens J. Sifrim D. (2003). Relevance of Ineffective Oesophageal Motility during Oesophageal Acid Clearance. Gut 52, 784–790. 10.1136/gut.52.6.784 12740331 PMC1773669

[B59] Smout A. Fox M. (2012). Weak and Absent Peristalsis. Neurogastroenterol. Motil. 24 (Suppl. 1), 40–47. 10.1111/j.1365-2982.2011.01831.x 22248107

[B60] Süto G. Czirják L. (2009). Oesophageal Involvement in Scleroderma. Clin. Exp. Rheumatol. 27 (3 Suppl. 54), 2–4. 19796553

[B61] Svendsen K. Wood M. Olsson E. Nordeng H. (2018). Reported Time to Onset of Neurological Adverse Drug Reactions Among Different Age and Gender Groups Using Metoclopramide: an Analysis of the Global Database Vigibase. Eur. J. Clin. Pharmacol. 74, 627–636. 10.1007/s00228-017-2407-z 29290074

[B62] Sweis R. Heinrich H. Fox M. (2018). Variation in Esophageal Physiology Testing in Clinical Practice: Results from an International Survey. Neurogastroenterol. Motil. 30, e13215. 10.1111/nmo.13215 28948708

[B63] Tack J. Camilleri M. Chang L. Chey W. D. Galligan J. J. Lacy B. E. (2012). Systematic Review: Cardiovascular Safety Profile of 5-HT4 Agonists Developed for Gastrointestinal Disorders. Aliment. Pharmacol. Ther. 35, 745–767. 10.1111/j.1365-2036.2012.05011.x 22356640 PMC3491670

[B64] Triadafilopoulos G. Tandon A. Shetler K. P. Clarke J. (2016). Clinical and pH Study Characteristics in Reflux Patients with and without Ineffective Oesophageal Motility (IEM). BMJ Open Gastroenterol. 3, e000126. 10.1136/bmjgast-2016-000126 PMC5174815 28074151

[B65] Tsai S. C. Kao C. H. Pan D. Y. ChangLai S. P. Wang S. J. (1995). Effects of Oral Erythromycin on Esophageal Motility in Patients with Noninsulin-dependent Diabetes Mellitus. Gaoxiong Yi Xue Ke Xue Za Zhi 11, 430–435. 7674422

[B66] Wang D. Wang X. Yu Y. Xu X. Wang J. Jia Y. (2019). Assessment of Esophageal Motor Disorders Using High-Resolution Manometry in Esophageal Dysphagia with normal Endoscopy. J. Neurogastroenterol. Motil. 25, 61–67. 10.5056/jnm18042 30646476 PMC6326201

[B67] Wang V. S. Feldman N. Maurer R. Burakoff R. (2009). Esophageal Motility in Nonacid Reflux Compared with Acid Reflux. Dig. Dis. Sci. 54, 1926–1932. 10.1007/s10620-008-0580-8 19051027

[B68] Wu J. C. Y. Cheung C. M. Y. Wong V. W. S. Sung J. J. Y. (2007). Distinct Clinical Characteristics between Patients with Nonerosive Reflux Disease and Those with Reflux Esophagitis. Clin. Gastroenterol. Hepatol. 5, 690–695. 10.1016/j.cgh.2007.02.023 17481961

[B69] Xiao Y. Kahrilas P. J. Nicodème F. Lin Z. Roman S. Pandolfino J. E. (2014). Lack of Correlation between HRM Metrics and Symptoms during the Manometric Protocol. Am. J. Gastroenterol. 109, 521–526. 10.1038/ajg.2014.13 24513804 PMC4120962

[B70] Xu J.-Y. Xie X.-P. Song G.-Q. Hou X.-H. (2007). Healing of Severe Reflux Esophagitis with PPI Does Not Improve Esophageal Dysmotility. Dis. Esophagus. 20, 346–352. 10.1111/j.1442-2050.2007.00681.x 17617885

[B71] Yi C.-H. Lei W.-Y. Hung J.-S. Liu T.-T. Chen C.-L. (2016). Effects of Prucalopride on Esophageal Secondary Peristalsis in Humans. Clin. Transl. Gastroenterol. 7, e202. 10.1038/ctg.2016.58 27831544 PMC5288569

